# Supplementary material for: A virtual screening and molecular dynamics approach in search of novel antibiotic chemotypes
Source: PLoS One. 2026 Mar 20;21(3):e0341835. doi: 10.1371/journal.pone.0341835 (PMC13004388; doi:10.1371/journal.pone.0341835)

**Supporting Information**

**Supplementary Figure 1:** Minimum Inhibitory Concentration (MIC), in µg/mL, of **8802** against selected bacterial strains


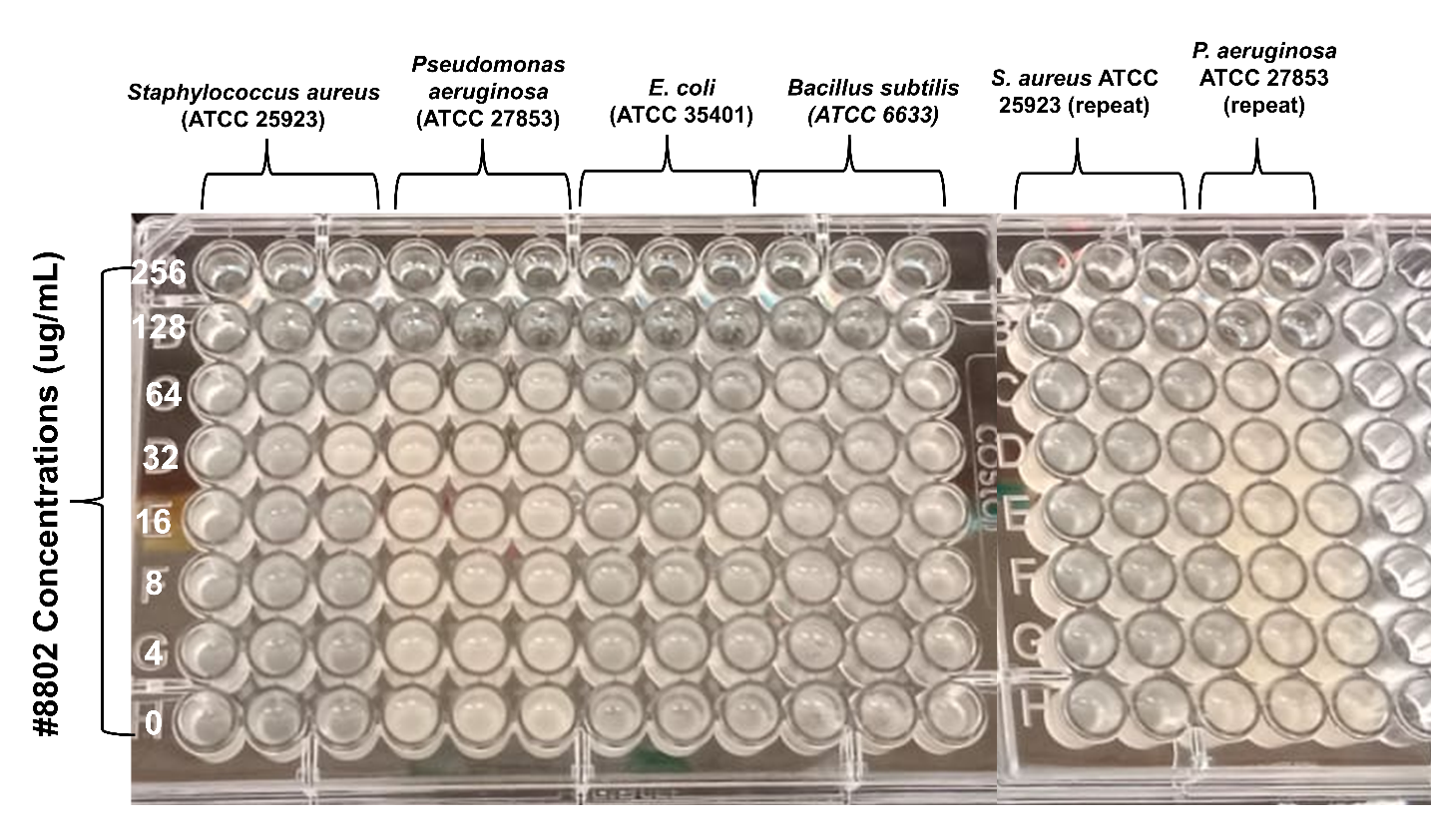

Supplement: S1 Fig — (DOCX) [file pone.0341835.s002.docx]
