# Supplementary material for: A virtual screening and molecular dynamics approach in search of novel antibiotic chemotypes
Source: PLoS One. 2026 Mar 20;21(3):e0341835. doi: 10.1371/journal.pone.0341835 (PMC13004388; doi:10.1371/journal.pone.0341835)
Supplement: S4 Fig — (DOCX) [file pone.0341835.s004.docx]

**Supporting Information**

**Supplementary Figure 4.** Minimum Inhibitory Concentration (MIC), in µg/mL, of **LST-2** against selected bacterial strains.

**
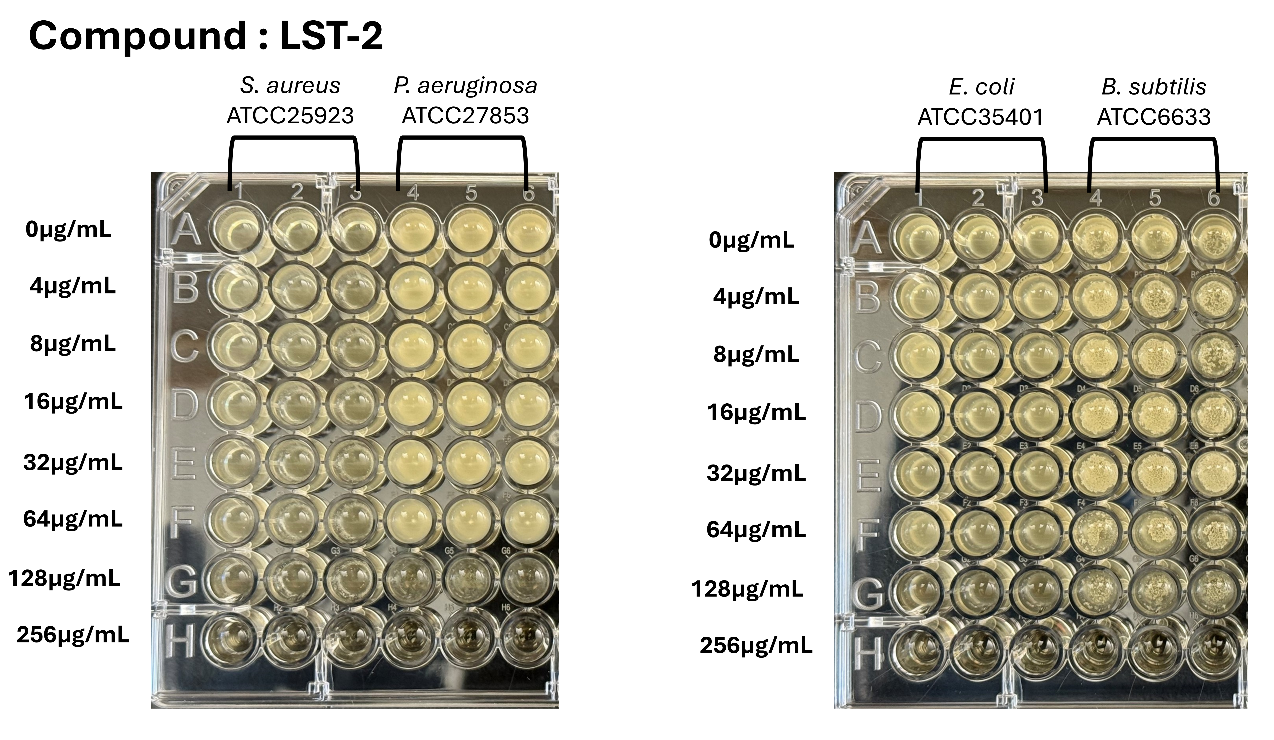
**
