# Supplementary material for: A virtual screening and molecular dynamics approach in search of novel antibiotic chemotypes
Source: PLoS One. 2026 Mar 20;21(3):e0341835. doi: 10.1371/journal.pone.0341835 (PMC13004388; doi:10.1371/journal.pone.0341835)
Supplement: S5 Fig — Concentration range: 10 nM – 50 µM. (DOCX) [file pone.0341835.s005.docx]

**Supporting Information**

**Supplementary Figure 5.** Positive Control SPR Curve for Compound Lee1532 (Compound **10**). Concentration range: 10 nM – 50 µM.

**
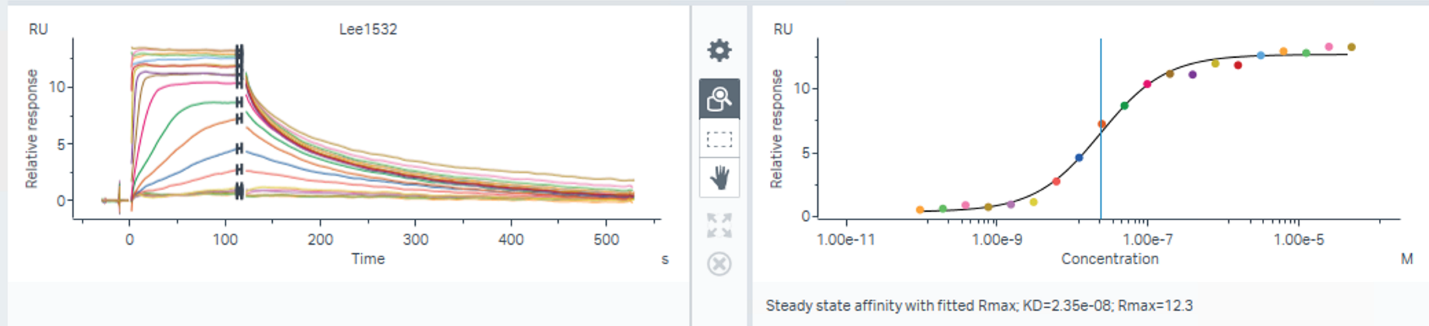
**
