# Supplementary material for: A virtual screening and molecular dynamics approach in search of novel antibiotic chemotypes
Source: PLoS One. 2026 Mar 20;21(3):e0341835. doi: 10.1371/journal.pone.0341835 (PMC13004388; doi:10.1371/journal.pone.0341835)
Supplement: S10 Fig — (DOCX) [file pone.0341835.s010.docx]

**Supporting Information**

**Supplementary Figure10.** ^1^H (400 MHz) and ^13^C NMR (151 MHz) spectra in DMSO-*d6* for **LST-1**.
