# Supplementary figures and images for: A virtual screening and molecular dynamics approach in search of novel antibiotic chemotypes
Source: PLoS One. 2026 Mar 20;21(3):e0341835. doi: 10.1371/journal.pone.0341835 (PMC13004388; doi:10.1371/journal.pone.0341835)

**Supporting Information**

**Supplementary Figure12.** RMSD of the **8802**-MtbDHPS complex from 0–110 ns.


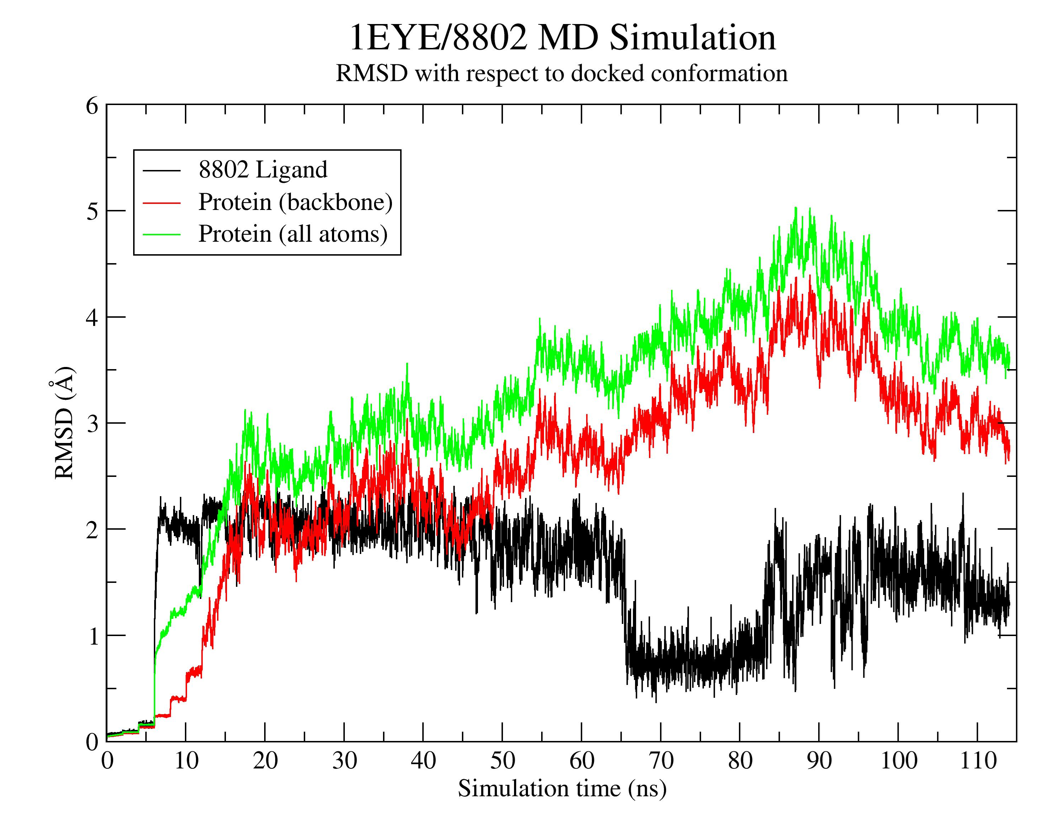

Supplement: S12 Fig — (DOCX) [file pone.0341835.s012.docx]

**Supporting Information**

**Supplementary Figure13.** RMSD of the **7034**-MtbDHPS complex from 0–110 ns.


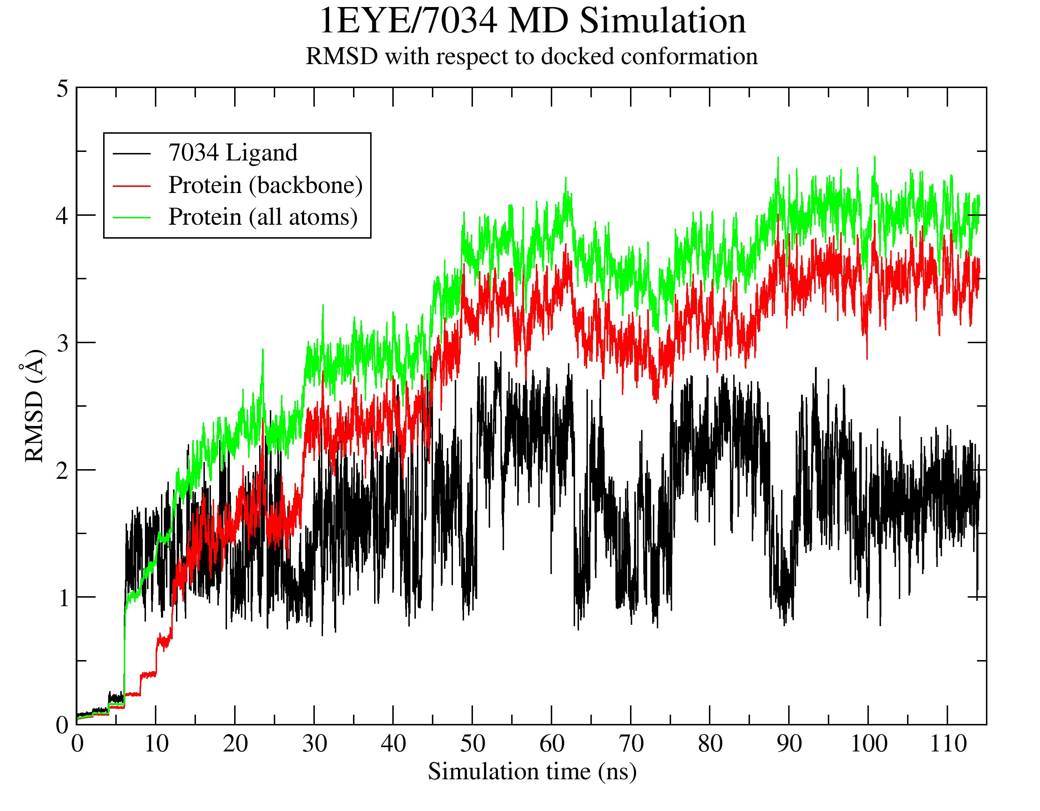

Supplement: S13 Fig — (DOCX) [file pone.0341835.s013.docx]

**Supporting Information**

**Supplementary Figure14.** RMSD of the **LST**-**1-**MtbDHPS complex from 0–110 ns.


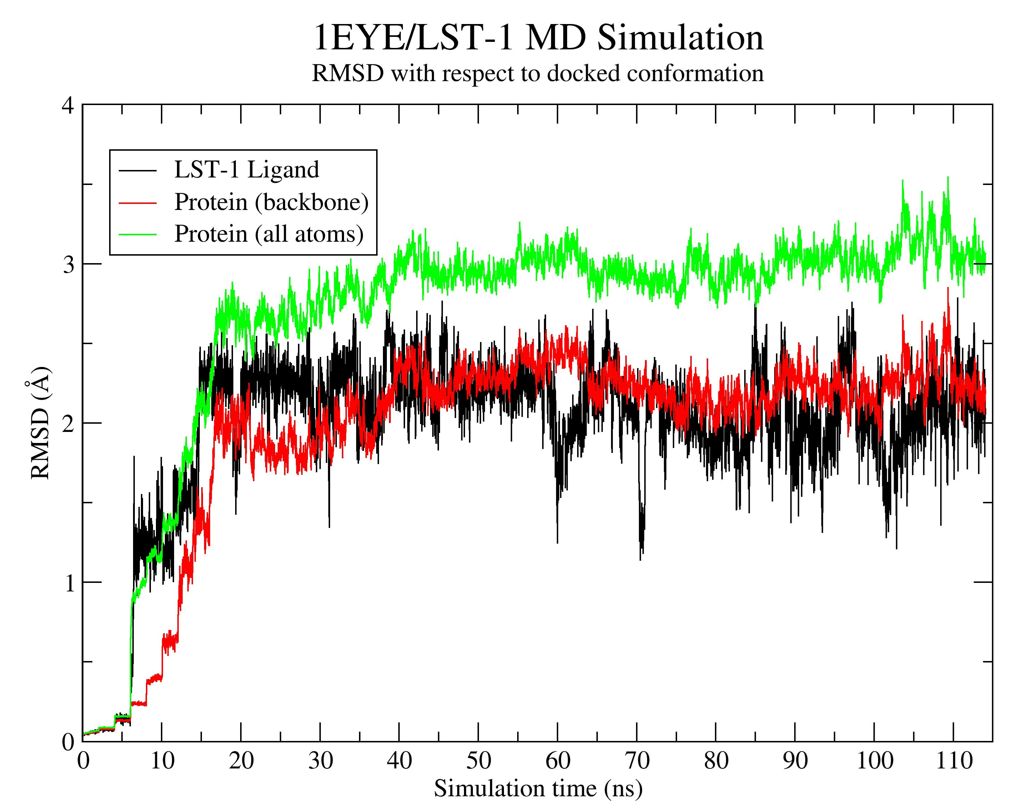

Supplement: S14 Fig — (DOCX) [file pone.0341835.s014.docx]

**Supporting Information**

**Supplementary Figure15.** RMSD of the **LST-2**-MtbDHPS complex from 0–110 ns.


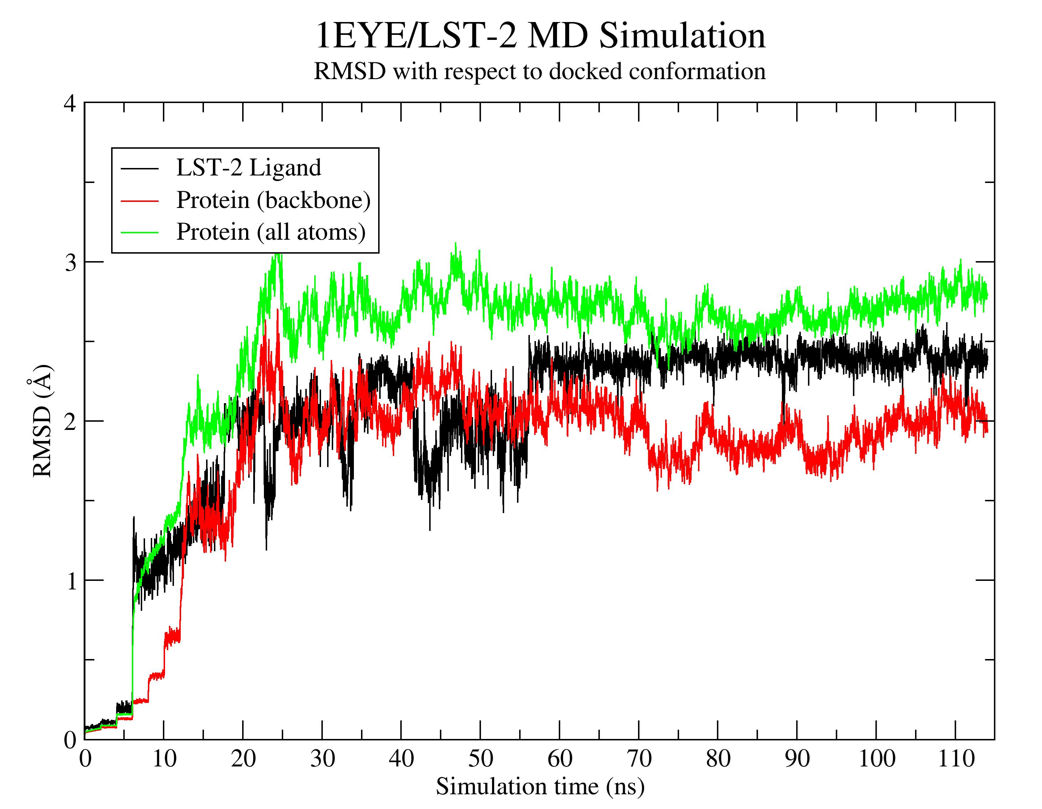

Supplement: S15 Fig — (DOCX) [file pone.0341835.s015.docx]
