# Supplementary material for: A virtual screening and molecular dynamics approach in search of novel antibiotic chemotypes
Source: PLoS One. 2026 Mar 20;21(3):e0341835. doi: 10.1371/journal.pone.0341835 (PMC13004388; doi:10.1371/journal.pone.0341835)
Supplement: Attachment — Submitted filename: Review Report (PONE-D-25-47619) .pdf [file pone.0341835.s016.pdf]

<u>Review Report (PONE-D-25-47619)</u>

The manuscript addresses an important problem of antibiotic resistance and reports the virtual screening, synthesis, docking, simulation and biological evaluation of novel DHPS inhibitors. The study is extensive, but several serious concerns must be addressed before the work can be considered for publication in PLOS ONE.

1. Rationale for compound selection is weak. Only 8802 and 7034 were chosen based on docking score and commercial availability. Authors must explain why other top hits were not considered.

2. Selection of PDB ID (1EYE, M.tb DHPS) is questionable given that antibacterial testing was done on *E. coli, P. aeruginosa, S. aureus,* and *B. subtilis*. A justification of homology and choice is required.

3. Discrepancy between in silico predictions and in vitro validation: Docking/MD suggests strong binding, but fluorescence polarization and SPR show weak or no binding. Authors must address this contradiction critically.

4. MIC assays lack clarity on replicates, statistical analysis, and controls. The use of FQ5 as a positive control is insufficient; more appropriate controls (e.g., sulfonamides, trimethoprim) should be included.

5. Discussion currently overstates the conclusions. The weak experimental validation must be reconciled with computational predictions rather than speculated upon.

1. Grammar requires professional English editing including overuse of adverbs (e.g., Interestingly, Gratifyingly).

2. Figures and tables formatting inconsistent. Some links to figures not properly embedded.

3. Reference style inconsistent with PLOS ONE format.

4. Abbreviations (e.g., DHPS, MolPSA) used without consistent definition.

Recommendation: Major Revision
