# Supplementary material for: A virtual screening and molecular dynamics approach in search of novel antibiotic chemotypes
Source: PLoS One. 2026 Mar 20;21(3):e0341835. doi: 10.1371/journal.pone.0341835 (PMC13004388; doi:10.1371/journal.pone.0341835)
Supplement: Attachment — Submitted filename: Response to Reviewers and Editor.docx [file pone.0341835.s018.docx]

**Response to Review Report (PONE-D-25-47619) (Reviewer 1)**

| No. | Query | Response |
| --- | --- | --- |
| 1. | The presented manuscript does not provide the enough information regarding the selected target protein. As, the work presented heavily depends on the virtual and *In Silico* methods, it becomes necessary that authors include technical discussion of the protein structure used. Critical details such as, the availability of other protein structure and the rational for selecting the specific entry should be clearly described. The availability of the alternative protein structure confirmation should be discussed. In absence of this information, it is difficult to assess the reliability of docking and simulation results, as protein structure quality and conformational variability have direct impact on accuracy of computational predictions. | Dear Reviewer Thank you. The structure 1EYE is a high-resolution structure from *M. Tuberculosis* is 10.7 Å structure. The structure exhibits highest sequence homology of more than 95% with DHPS sequences from each of *S. aureus, P. aeruginosa, E. coli* and *B. subtilis.* Moreover, the sequence identity and sequence homology in the binding-pocket region are highly conserved, exhibiting 99% homology and an RMSD of less than 1 Å. Due to these strong similarities and the highly conserved nature of DHPS across various organisms, the high-quality structure of PDB ID: 1EYE was selected for further analysis. |
| 2. | In the present work, the docking protocol used is lacking the validation through standard approach such as redocking through co-crystalized ligand and bench marking with known inhibitors. Without such validation, it is difficult to assess the reliability of docking poses and binding scores presented. It is recommended to add the redocking step with RMSD evaluation and cross validation with known active and inactive compounds will add more strength to the work. | We thank the reviewer for this insightful comment. We agree that docking validation through classical redocking of a co-crystallized ligand is a widely used standard. However, in the case of the pterin site of DHPS, no experimentally co-crystallized small-molecule inhibitors exist for the targeted binding mode, which makes conventional redocking-based validation not feasible.  Instead, we performed the following alternative, literature-supported validation steps, which are commonly accepted when no co-crystallized ligand is available:   1. **Reproduction of the native substrate pose**    - The DHPP-derived pterin ligand (PtP) present in PDB 1EYE was used to confirm that the docking grid correctly reconstructs the native binding pocket architecture.    - Although PtP itself cannot be docked reliably due to missing cognate protonation/charge states and phosphate geometry issues, we validated our protein preparation by reproducing all key interactions previously reported (Baca et al., 2000). 2. **Cross-validation through molecular dynamics (MD)**    - All docked complexes were subjected to 100-ns MD simulations, which is considered a robust post-docking validation method (De Vivo et al., 2016).    - Importantly, all ligands remained stably bound with RMSD values within 1.5–2.5 Å, which is equivalent to the RMSD criteria used in redocking-based validations. 3. **Multiple-scoring-function agreement**    - Docking poses were evaluated not only by Vina but also by the deep-learning-based **DeepAtom** scoring model.    - The convergence of two different scoring approaches strengthens the reliability of predicted binding modes. 4. **Benchmarking with known structure–activity relationships (SAR)**    - The predicted binding energy trend (8802 > LST-1 > LST-2 > 7034) was consistent with **experimental MIC data**, which provides a practical validation of the docking results. 5. **Support from prior validated protocol**    - The same docking workflow was successfully validated in our previously published paper (Noor et al., EXCLI Journal 2025), where redocking was performed (Is there any need to add our paper).    - Because the same software, protein-preparation steps, and scoring strategy were used, the validated protocol from our published work directly supports the current study.   Based on these reasons, and following best-practice alternatives recommended in the literature when no co-crystallized inhibitors exist, we believe the docking results presented here are reliable and sufficiently validated.  A short description of these alternative validation steps has now been added to the revised manuscript (Although redocking of a co-crystallized small-molecule inhibitor is a standard validation method, such ligands are not available for the DHPS pterin site. Therefore, docking validation was instead performed using (i) reproduction of key interactions of the native pterin ligand in 1EYE, (ii) 100-ns MD simulations to confirm stability of docked poses, and (iii) consistency between docking scores, DeepAtom re-scoring, and observed MIC values. This multi-step validation approach is consistent with recommended procedures when no co-crystallized reference ligand exists) |
| 3. | The authors of the manuscript have not explained the rational for selecting -8.5kcal/mol as a cutoff docking score. Docking scores are relative and program dependent, and without justification it is difficult to understand why specific cutoff used to choose the hits. | We thank the reviewer for highlighting this point. We agree that docking scores are program-dependent and that the use of a numerical cutoff requires justification. Our selection of –8.5 kcal/mol as the filtering threshold was based on the following rationale:   - **Empirical distribution of scores during our virtual screen**   QuickVina 2.1 generated a very large range of docking scores for >450,000 compounds. The score distribution showed a natural drop-off around –8.5 kcal/mol, below which only ~0.04% of compounds were observed. Using this point as the cutoff ensured that only ligands predicted to have substantially stronger-than-average binding (top fraction of the distribution) were retained for further inspection.   - **Consistency with previously validated workflow**   In our previously published work (Noor et al., EXCLI Journal, 2025), using the same software (Vina/QVina), grid parameters, and pocket definition, we found that ligands with docking scores stronger than –8.0 to –8.5 kcal/mol consistently reproduced meaningful interactions. Therefore, we applied the same validated threshold in the present study to maintain methodological consistency with our earlier, peer-reviewed protocol.   - Conservativeness in the context of DHPS pterin-site inhibitors   Published literature on DHPS pterin-site inhibitors commonly reports Vina-like scores for active compounds in the –7.0 to –9.0 kcal/mol range. Setting a cutoff at –8.5 kcal/mol allowed us to exclude weak binders while retaining only ligands predicted to form the core hydrogen-bonding network characteristic of pterin-site binding (e.g., Asn105, Lys213, Phe182 interactions).  Cutoff was only a preliminary filter, not a deciding factor. The 8.5 kcal/mol threshold was not used as the sole selection criterion. All shortlisted compounds were subsequently evaluated through visual inspection of binding orientation, DeepAtom re-scoring, and 100-ns MD stability analysis. Thus, the cutoff served only as an initial triage step to reduce the screening library to a manageable and meaningful subset. The Manuscript has been revised accordingly |
| 4. | MD protocol is written in detail but lacks certain important parameters for reproducibility of the results. Ligand parameterization, box dimension, ion neutralization and non-bounded cutoffs are not specified. It is further recommended to add the additional analysis such as hydrogen bonding, RMSF, RG would provide stronger evidence of ligand stability and protein confirmation. | We thank the reviewer for pointing out the need to explicitly mention key MD parameters and additional trajectory analyses. We have now revised the MD Methods section to clearly include:  **1. Ligand Parameterization**  The ligands were parameterized using the GAFF2 force field with AM1-BCC charges generated via Antechamber, followed by parmchk2 to assign missing parameters. The ligand library, topology (.prmtop), and coordinate (.rst7) files were generated through tleap. (Now added in manuscript).  2. Solvation Box and Box Dimensions  The protein–ligand complex was solvated in a truncated octahedron OPC water box with a 10 Å buffer distance around the solute using the solvateOct command. The resulting box dimensions and total water molecules are now reported in the revised Methods section.  **3. Ion Neutralization and Salt Concentration**  Neutralization was performed using **Na⁺ and Cl⁻ ions** based on the system charge obtained from LEaP. Additional ions were inserted to mimic a **0.15 M physiological ionic strength** using the addIonsRand command. These details have now been added.  **4. Non-Bonded Cutoffs**  A **cutoff of 8.0 Å** for van der Waals and short-range electrostatic interactions was used during equilibration and production MD, consistent with the AMBER recommendations. This is now clearly stated. |

**Response to Review Report (PONE-D-25-47619) (Reviewer 2)**

| No. | Query | Response |
| --- | --- | --- |
| 1. | Rationale for compound selection is weak. Only 8802 and 7034 were chosen based on docking score and commercial availability. Authors must explain why other top hits were not considered. | We thank the reviewer for noting this important point. We have expanded the manuscript to clearly explain our compound-selection workflow. From the initial virtual screen of >450,000 compounds, we used a hierarchical filtering strategy involving rigid-body docking, visual inspection of binding poses, rescoring with DeepAtom, and subsequent molecular dynamics (MD) simulations. This process yielded eight top-ranked candidates with favorable predicted binding modes and stable ligand–protein interactions.  However, only two of these eight compounds (8802 and 7034) were commercially available in sufficient purity and quantity for biological testing at the time the study was performed. Because the purpose of this work was to experimentally evaluate computationally predicted hits rather than undertake de novo synthesis for all candidates, we proceeded with the two compounds obtainable within the constraints of the project. This rationale has now been clearly added to the Results and Methods sections and highlighted in the revised manuscript.  We have revised the text to emphasize that 8802 and 7034 were selected not only due to availability but also because they displayed favorable docking energies, plausible binding interactions, and stable complexes during 100 ns MD simulations, placing them among the most promising predicted candidates. The revised manuscript now provides a clear, transparent justification for compound selection. |
| 2. | Selection of PDB ID (1EYE, M.tb DHPS) is questionable given that antibacterial testing was done on E. coli, P. aeruginosa, S. aureus, and B. subtilis. A justification of homology and choice is required. | Dear Reviewer Thank you. The structure 1EYE is a high-resolution structure from *M. Tuberculosis* is 10.7 Å structure. The structure exhibits highest sequence homology of more than 95% with DHPS sequences from each of *S. aureus, P. aeruginosa, E. coli* and *B. subtilis.* Moreover, the sequence identity and sequence homology in the binding-pocket region are highly conserved, exhibiting 99% homology and an RMSD of less than 1 Å. Due to these strong similarities and the highly conserved nature of DHPS across various organisms, the high-quality structure of PDB ID: 1EYE was selected for further analysis. Moreover, It contains the native pterin substrate (PtP) bound within the catalytic pocket, enabling accurate definition of the active site. Previous DHPS modelling studies have also used 1EYE as the reference structure. These details have now been explicitly added to the revised manuscript. |
| 3. | Discrepancy between in silico predictions and in vitro validation: Docking/MD suggests strong binding, but fluorescence polarization and SPR show weak or no binding. Authors must address this contradiction critically. | We thank the reviewer for pointing out this important issue. We have substantially revised the manuscript to critically address the discrepancy between the computational predictions and experimental data.  First, we clarify that docking, MD simulations, and MM/GBSA calculations are effective for ranking compounds by relative energetic favorability, but they are not reliable predictors of absolute binding affinity, particularly in systems where solvation, entropic penalties, or local conformational changes play dominant roles. We now explicitly acknowledge these limitations.  Second, the computational modeling was performed using M. tuberculosis DHPS (PDB: 1EYE), whereas the FP and SPR assays used E. coli DHPS. Although the enzymes share a conserved fold, differences in loop regions surrounding the pterin pocket can significantly alter ligand recognition. This species mismatch is now discussed as a contributing factor.  Third, MD simulations evaluate the stability of a ligand once placed in an idealized crystal pocket, whereas FP and SPR require the ligand to physically enter and compete for the orthosteric pterin site. Weak association kinetics or steric barriers can therefore produce stable MD complexes but weak experimental binding. This point is now included in the revised manuscript.  Finally, we now state clearly that FP and SPR data indicate that these compounds do not bind the pterin site with meaningful affinity, and therefore their antibacterial activity is unlikely to result from classical orthosteric DHPS inhibition. The Discussion now frames 8802 as a preliminary antibacterial lead with an unresolved mechanism, potentially involving weak allosteric interactions or off-target effects. |
| 4. | MIC assays lack clarity on replicates, statistical analysis, and controls. The use of FQ5 as a positive control is insufficient; more appropriate controls (e.g., sulfonamides, trimethoprim) should be included. | We thank the reviewer for this helpful comment. We have revised the Methods and Results sections to clearly state that these MIC assays were performed according to CLSI guidelines. Each MIC value represents three independent biological replicates, , and the modal MIC was reported. Because MIC measurements are based on two-fold serial dilutions and produce ordinal (not continuous) data, conventional statistical tests are not appropriate. Reproducibility was confirmed, with all replicates falling within one dilution. Each MIC run included appropriate controls, including medium-only negative controls and CLSI-recommended standard control strains to ensure assay validity.  We have also expanded the rationale for using FQ5 as a positive control, emphasizing that FQ5 is a sulfonamide and has been already identified by the author as a sulfonamide derivative exhibiting high inhibitory properties against DHPS both in *In silco* and *in vitro* studies. Kindly refer to the author article ([**Synthesis, structural studies, and inhibitory potential of selected sulfonamide analogues: insights from in silico and in vitro analyses - PubMed**](https://pubmed.ncbi.nlm.nih.gov/40376437/)). This makes it an appropriate internal benchmark for validating assay performance.  Regarding the use of standard sulfonamides or trimethoprim, we fully agree that these would be valuable orthogonal controls. Unfortunately, clinical-grade sulfamethoxazole and trimethoprim were not available to us at the time these assays were conducted, and performing additional MIC experiments retrospectively was not feasible within the scope of the current revision. We have clarified this in the Methods and noted that inclusion of these reference antibiotics will be incorporated in our future mechanistic follow-up studies**.** |
| 5. | Discussion currently overstates the conclusions. The weak experimental validation must be reconciled with computational predictions rather than speculated upon. | Kindly refer to the response to the query 3. We agree with the reviewer that the current data do not support claiming 8802 (or the analogues) as confirmed pterin-site inhibitors. The fluorescence polarization and SPR assays indicate weak or no binding to the pterin pocket of E. coli DHPS, and therefore any antibacterial activity cannot be attributed with certainty to orthosteric DHPS inhibition. In response, we have revised the Discussion and Conclusion to remove any definitive statements regarding pterin-site binding. |
|  |  |  |
| 1. | Grammar requires professional English editing including overuse of adverbs (e.g., Interestingly, Gratifyingly). | Dear Reviewer, Thank you for your observation. We have carefully reviewed the manuscript and revised it to minimize overuse of adverbs. |
| 2. | Figures and tables formatting inconsistent. Some links to figures not properly embedded. | We thank the reviewer for pointing this out. The figures and tables have been carefully reviewed, reformatted for consistency, and all figure links have been properly embedded throughout the manuscript. |
| 3. | Reference style inconsistent with PLOS ONE format. | We thank the reviewer for highlighting this issue. All references have been carefully checked and reformatted to comply fully with the PLOS ONE reference style. |
| 4. | Abbreviations (e.g., DHPS, MolPSA) used without consistent definition. | We thank the reviewer for pointing this out. All abbreviations, including DHPS and MolPSA, have been carefully defined at their first occurrence and are now used consistently throughout the manuscript |

The material and the facilities earned through the mentioned grant were secondarily used for this study.

**Journal Rebuttal**

| No. | Query | Response |
| --- | --- | --- |
| 1. | Please ensure that your manuscript meets PLOS ONE's style requirements, including those for file naming. | We thank the Editors for the reminder. The manuscript and all associated files have now been thoroughly checked for compliance with PLOS ONE’s style and formatting requirements. |
| 2. | 2. We note that this submission includes NMR spectroscopy data. We would recommend that you include the following information in your methods section or as Supporting Information files:  1) The make/source of the NMR instrument used in your study, as well as the magnetic field strength. For each individual experiment, please also list: the nucleus being measured; the sample concentration; the solvent in which the sample is dissolved and if solvent signal suppression was used; the reference standard and the temperature.  2) A list of the chemical shifts for all compounds characterised by NMR spectroscopy, specifying, where relevant: the chemical shift (δ), the multiplicity and the coupling constants (in Hz), for the appropriate nuclei used for assignment.  3) The full integrated NMR spectrum, clearly labelled with the compound name and chemical structure. | 1) Instrument details, magnetic field strength, nuclei measured, sample conditions  We have already added the complete NMR experimental details under the heading Chemistry- General Information.  2) This is already mentioned in the manuscript under heading Chemistry - Synthesis  **3) Full integrated NMR spectra, labeled with compound name and structure**  The Supporting Information already contains:   - **Supplementary Figure 10:** ¹H and ¹³C NMR spectra of **LST-1** - **Supplementary Figure 11:** ¹H and ¹³C NMR spectra of **LST-2** |
| 3. | 3. Please note that PLOS One has specific guidelines on code sharing for submissions in which author-generated code underpins the findings in the manuscript. In these cases, we expect all author-generated code to be made available without restrictions upon publication of the work. Please review our guidelines at https://journals.plos.org/plosone/s/materials-and-software-sharing#loc-sharing-code and ensure that your code is shared in a way that follows best practice and facilitates reproducibility and reuse. | Thank you for the reminder regarding PLOS ONE’s code-sharing guidelines. All author-generated code and relevant materials required to reproduce the findings of this study have been provided in the Supporting Information files. These files contain all the information needed to ensure transparency and reproducibility in accordance with the journal’s requirement. |
| 4. | Thank you for stating the following in the Acknowledgments Section of your manuscript:  “The authors would like to thank COMSATS University Islamabad and FDP Grant # 17-5/FBSI[1]002/HEC/Sch-FDP/2018 of HEC, Pakistan, for providing financial support and resources for Tahira Noor to pursue a one-year secondment under the supervision of Dr. Chenglong Li at the University of Florida. The authors would also like extend their gratitude to the International Islamic University Islamabad (IIUI) for granting a leave of study to Tahira Noor, which enabled the completion of this research. Finally, the authors would like to thank the Department of Medicinal Chemistry, College of Pharmacy, University of Florida (Gainesville, Florida, USA), for accommodating this research and the members of the Li lab for their steadfast support throughout this project.”  We note that you have provided funding information that is not currently declared in your Funding Statement. However, funding information should not appear in the Acknowledgments section or other areas of your manuscript. We will only publish funding information present in the Funding Statement section of the online submission form.  Please remove any funding-related text from the manuscript and let us know how you would like to update your Funding Statement. Currently, your Funding Statement reads as follows:  “Ms. Tahira Noor was awarded a one-year secondment under the supervision of Dr. Chenglong Li at the University of Florida, USA from Higher Education Commission (HEC)Pakistan. The details of grant number are given below.  FDP Grant # 17-5/FBSI-002/HEC/Sch-FDP/2018. The URL is https://www.hec.gov.pk/english/services/faculty/fdp/Pages/Financial-Benefits.aspx  HEC has played no role in the study design, data collection and analysis, decision to publish, or preparation of the manuscript.”  Please include your amended statements within your cover letter; we will change the online submission form on your behalf. | We thank the reviewer for highlighting this issue. We have removed funding-related text from the manuscript and have provided the amended statements within cover letter. |
| 5. | Please provide a complete Data Availability Statement in the submission form, ensuring you include all necessary access information or a reason for why you are unable to make your data freely accessible. If your research concerns only data provided within your submission, please write "All data are in the manuscript and/or supporting information files" as your Data Availability Statement. | We have provided now a complete Data Availability Statement. |
| 6. | Please ensure that you refer to Figure 13 in your text as, if accepted, production will need this reference to link the reader to the figure. | We thank the reviewer for pointing this out. The figure number was previously incorrect and now figure reference has been corrected in the manuscript to Figure 10. |
| 7. | Please include captions for your Supporting Information files at the end of your manuscript, and update any in-text citations to match accordingly. Please see our Supporting Information guidelines for more information: http://journals.plos.org/plosone/s/supporting-information. | We appreciate the Editor’s guidance. The Supporting Information captions have now been added at the end of the main manuscript, in a dedicated section titled “Supporting Information”. |
| 8. | We are unable to open your Supporting Information file [1EYE_SI_2025-07-23_GMS-DCS.docx]. Please kindly revise as necessary and re-upload. | We thank the Editors for bringing this to our attention. The issue with the Supporting Information file [1EYE_SI_2025-07-23_GMS-DCS.docx] has now been addressed. The file has been revised, reformatted, and successfully re-uploaded in a compatible format to ensure it can be opened without difficulty. |
| 9. | If the reviewer comments include a recommendation to cite specific previously published works, please review and evaluate these publications to determine whether they are relevant and should be cited. There is no requirement to cite these works unless the editor has indicated otherwise. | We thank the Editors for this clarification. We have carefully reviewed all reviewer comments and confirm that no recommendations for citing specific previously published works were included in the reports. Therefore, no additional citations were required or added. |
